# Supporting implementation of Cochrane methods in complex communication reviews: resources developed and lessons learned for editorial practice and policy

**DOI:** 10.1186/s12961-019-0435-0

**Published:** 2019-03-28

**Authors:** Rebecca Ryan, Sophie Hill

**Affiliations:** Cochrane Consumers and Communication Group, Centre for Health Communication and Participation, School of Psychology and Public Health, College of Science, Health and Engineering, La Trobe University, Victoria, 3086 Australia

**Keywords:** Evidence-based practice, Health communication, Health services research, Patient participation

## Abstract

Every healthcare encounter involves some form of communication and there is growing recognition that effective health communication is central to the delivery of safe, high-quality healthcare. Conversely, poor communication has a range of adverse consequences for those receiving healthcare and the systems delivering care, including elevated patient safety risks. Increasing understanding and documentation of the key role that good communication plays in healthcare design and delivery has meant there is growing demand from policy-makers and other decision-makers for evidence on the effects of health communication interventions – that is, how best to communicate. While systematic reviews of such interventions are fundamental to building this evidence base, such interventions and reviews are often highly complex and pose considerable challenges for authors and editors. In this paper, we describe our experience as a Cochrane editorial group identifying common issues in reviews of communication interventions and developing resources to support authors to better meet these challenges. Our analysis found that issues typically fell into one or more of the following three stages of the review process: understanding and applying systematic review methods (e.g. selecting outcomes for analysis); reporting the review’s methods (e.g. describing key decisions made in conducting the review); and interpreting the findings (e.g. incorporating quality of the evidence into findings of the review). We also found that common issues reflected both practical difficulties (such as the typically large size of reviews and disparate measures for outcomes) and conceptual challenges (for instance, the difficulties of identifying comparisons). While extensive advice for Cochrane systematic reviewers exists, this standardised advice does not cover all of the issues emerging for complex communication reviews. In response, we therefore developed a collection of resources, both general and targeted to specific methodological issues. Here, we describe the types of resources developed and the aims of these, the rationale for why we needed to fill specific gaps in existing advice, and reflect on the lessons for future editorial practice, policies and research in relation to the implementation of Cochrane review methods in the area of health communication.

## Background

There is increasing recognition that systematically reviewing the evidence on the effects of complex interventions presents a set of unique challenges [1–3]. Many definitions exist, most indicating that a complex intervention includes several components, involves multiple causal pathways, targets a range of different participants or organisational levels, and interacts with context in various ways [1–4]. Such features, amongst others, introduce complexity over the lifespan of a systematic review, from conception to interpretation and application of findings [1, 3, 5].

Guidance published in 2017 on complex interventions has aided systematic reviewers [1, 6–9]. Paradoxically, this highlights that advice is often produced many years after the problems were first identified [10–12], while in the interim editors and authors must deal with the difficulties of applying the existing advice.

For journal editors, this time lag presents challenges. This is particularly the case in Cochrane, an international organisation responsible for 15% of all of the world’s systematic reviews [13].

The Cochrane Consumers and Communication Group (CCCG) coordinates the preparation and production of reviews of interventions that influence the way that people interact with healthcare professionals, services and researchers (http://cccrg.cochrane.org). Collectively, we refer to these interventions as ‘health communication and participation’ interventions. At the time of writing, the Group’s portfolio includes 63 reviews and 30 protocols. The scope of most CCCG reviews is oriented to the effects of interventions on patients or carers regardless of their condition, ensuring the evidence is built across disease silos or health systems (e.g. [14, 15]) and reflecting the ubiquitous nature of health communication. Since every healthcare encounter involves some sort of communication, there is today widespread acknowledgement that effective communication is critical to the delivery of safe, high quality healthcare [16–21]. However, the converse is also true and increasingly well-documented – poor communication has demonstrable adverse consequences that affect the delivery and receipt of healthcare, and in particular, presents serious and imminent risks to patient safety. Communication breakdowns or failures happen in all healthcare settings and disciplines [16, 22–24], leading to poor outcomes such as medication errors of various sorts, increased hospital readmissions, emergency department presentations and longer hospital stays, and lack of follow-up care, including follow-up of abnormal test results or referrals. Such failures of communication and consequent adverse influences on patient safety are common and cumulative, and lead to considerable negative impacts for patients and health systems, including increases in mortality and serious adverse events, delayed treatment, intervention or diagnosis, treatment non-adherence, receipt of inappropriate or contraindicated treatments, and others such as psychological harms, uncertainty and inconvenience [18, 19, 22, 23, 25, 26]. With growing recognition and documentation of the essential role of good communication in healthcare design and delivery, policy-makers, safety and quality agencies, and health services are therefore looking to implement strategies about how best to communicate [27]. Systematic reviews evaluating the effects of communication interventions, such as those produced by CCCG, represent a critical component in building a high-quality evidence base on which such decisions about policy and practice rest.

The CCCG’s review portfolio, however, exemplifies many of the difficulties – for authors and editors alike – of implementing the standardised Cochrane systematic review methods in reviews of interventions in a socially and behaviourally complex area [28]. Conceptual difficulties abound, for example, populations may be socially (e.g. [29, 30]) or clinically (e.g. [14, 31]) constituted for the purpose of the review but may still be very diverse. Interventions may be direct and in-person (e.g. styles of communication [15, 32–34]), indirect (e.g. training [35, 36]), mediated by communication technology (e.g. automated phone calls [37], audio-visual information [38], electronic health records [39]), or publicly focussed (e.g. community-based or mass media-based education [40, 41]). Outcomes are measured with patients, health professionals or health services. For each, the range is considerable, such as patient knowledge, decision-making or behaviour related to health status or health service use [42]. In many areas there is no consensus about the definition of an intervention and its constituent parts, or on associated outcomes and their measurement [34, 43]. Compounding this, the definitions in use are highly fluid [29, 43, 44]. In this context, selecting ‘like’ interventions or identifying comparisons is difficult.

Additionally, the policy focus in recent decades on patient-centred care, patient experience or patient involvement [16, 21, 45–50] has meant that trials relevant to CCCG’s scope have increased rapidly. From a purely practical standpoint, many reviews are therefore becoming increasingly large and unwieldy (sometimes within the lifetime of one update), adding further difficulties for authors and editors [33, 34]. Editorial experience at the CCCG has shown that both practical and conceptual challenges are common and that these create significant methodological complexity but also barriers to timely completion of reviews and their updating [28].

The Cochrane model is one of encouraging wide participation in the conduct of reviews [51], with decentralised editorial support, core training and standardised methodological advice. The Cochrane Handbook underpins the methods and conduct of all reviews and, in the context of wide global participation, serves a critical educational purpose [52]. Despite this global support, in this editorial context, the CCCG observed frequent common errors across reviews. In common with other Cochrane Groups with a broad scope (e.g. the Effective Practice and Organisation of Care (EPOC) (https://epoc.cochrane.org) and Cochrane Infectious Diseases (https://cidg.cochrane.org) groups), we had a history of developing tailored advice to help authors implement methods in their reviews. In this commentary, we provide a summary of the resources developed, the rationale for why we needed to fill specific gaps, and reflect on the lessons for future editorial policies and practice.

## Methods

The CCCG identified two types of problems, namely (1) those intrinsic to the scope, diversity and fluidity of complex interventions and the consequent methodological issues; and (2) those intrinsic to author teams and their level of skills. This commentary focuses on the first problem, given the priority of methodological challenges in the ‘real-time’ context of editorial work for policy-relevant reviews of the evidence on communication.

Problem identification began systematically in 2009 but intensified following the formal introduction of the Cochrane Risk of Bias (RoB) tool [53], adoption of The Grading of Recommendations Assessment, Development and Evaluation (GRADE) system and Summary of Findings (SoF) tables [54], and the introduction (2016) and revision (2017) of the Methodological Expectations for Cochrane Intervention Reviews (MECIR) [55]. These evolutions in methods represent significant developments with global impact and a growing sophistication and application of systematic review methods, but they also imply, indirectly, a growing educational challenge when implementing the methods.

The editorial process for CCCG is as follows: all protocols and reviews are discussed and assessed by internal CCCG editors for quality prior to being sent to external peer review. The internal editorial group meets fortnightly and includes the Coordinating and Deputy Coordinating Editors, Managing Editor, Technical Editor and Information Specialist. Quality assessment actions are then determined and routinely include editorial review of all methods, and checks of the accuracy of data extraction from trials, RoB assessments, GRADE ratings of certainty and SoF tables. Issues which have the potential to most seriously distort the review’s findings receive the most attention. Standardised forms are used for accountability, record keeping and reporting back to author teams.

Identification and monitoring of problems as part of our quality assurance pathways found issues with the implementation of both established and newer methods. This mirrors other streams of work across Cochrane, which have identified clusters of commonly recurring problems across reviews, whether or not the interventions under evaluation are especially complex [56, 57]. Many problems commonly identified in CCCG reviews occurred despite the availability of relevant guidance such as that in the Cochrane Handbook [52]. Editorially, this indicated that applying standardised advice to reviews of socially complex interventions was often difficult for authors. In other cases, we noted gaps in the core advice available. For example, although there is much guidance to support the conduct of meta-analysis, advice to support synthesis without pooled analyses (instead using narrative synthesis, or a combination of meta-analysis and narrative synthesis) has lagged seriously behind, such that there was little formalised guidance available to support authors.

For each commonly occurring problem, we discussed whether a general or a more tailored resource was needed. In each case, we reviewed the availability and adequacy of existing advice for authors, comparing available resources with the common problems seen editorially. In drafting advice for each resource, we drew from the Cochrane Handbook [52] and advice from relevant methodological groups (e.g. GRADE [54], Template for intervention description and replication (TIDieR) [58], EPOC), and sought input from our wider editorial group, methodological experts or senior editors in the Cochrane Editorial and Methods Department (formally Cochrane Editorial Unit). All resources are made available on the CCCG website (http://cccrg.cochrane.org/author-resources), and we provide further tailored advice to author teams in the context of peer and editorial review processes. We seek feedback from authors on the resources, collect download statistics since July 2018, and aim to revise resources to improve usability and to accommodate changing methods and standards as they emerge.

## Results

First, we developed generic protocol and review templates which are provided to authors when titles are registered or the protocol is published (see Table 1 for all resources, their content and purpose). The purpose of the templates was to overcome the problem of providing the same advice repeatedly to teams about both basic and more complex methodological decisions. From our editorial perspective, it appeared that authors often had difficulty integrating and interpreting the available methodological advice from different sections or sources of guidance, or that applying standard advice to studies of socially complex interventions and phenomena is especially challenging. The templates were tailored to the scope of CCCG interventions. For instance, step-wise advice is given, with examples, on how to identify the range of relevant outcomes as well as on their categorisation and treatment for the purposes of analysis, and authors are prompted to consider how to identify groupings of complex interventions and appropriate comparison groups. Additionally, we developed and encouraged authors to use or adapt a generic data extraction template. This is a comprehensive template with numerous fields to capture the diversity of interventions for communication and participation and their key features. It is based on the TIDieR guidance [58] and on empirical research by members of the CCCG [59, 60]. Its main purpose was to encourage authors to collect sufficient information about included interventions to enable meaningful groupings for the purposes of analysis and synthesis, and to ensure that interpretation of the findings was based on an understanding of the effects of the interventions in context.

**Table 1:** Cochrane Consumers and Communication Group (CCCG) resources, content and purpose

Resource		Content	Purpose (problem addressed), examples
General resources that apply across review sections	CCCG protocol and review templates with guidance [69, 70]	Standardised text on the review methods (for adaptation) for use in RevMan [71] Identifies key issues associated with implementing the methods within each section of the protocol and review Provides reference to relevant sections of the Cochrane Handbook [52], to foundational documents (CCCG Study quality guide, CCCG Study Design guide) with cross-referencing to more specific CCCG supplemental guides	To support the application and reporting of methods in protocols and reviews Understanding and applying methods, for example: • Consistently rating risk of bias domains • Selecting outcomes for analysis Reporting methods, for example: • Describing methods and decisions adequately for replication • Reporting decisions related to grouping of studies and analyses transparently
	Data extraction template [72]	For use as a basis for planning the data extraction stage of reviews, and for piloting and adaptation to specific review content areas as required Template builds on research and earlier CCCG versions, Methodological Expectations for Cochrane Intervention Reviews (MECIR) standards [55] and other standards such as TIDieR (Template for intervention description and replication) [58]	To improve reporting of methods, intervention, other key component features, and findings and so support extraction of data that is meaningful for interpreting the findings of the review Reporting review methods, for example: • Describing methods and decisions adequately for replication • Justifying inclusion of additional (non-randomised controlled trial (RCT)) study designs • Acknowledging changes from protocol methods, including changes to selection criteria Interpreting findings, for example: • Considering quality of evidence alongside findings • Considering size, precision and direction of effects as well as results of tests of statistical significance
Resources with a specific focus	Short supplemental guides	Guides on specific issues commonly found to be problematic for authors in the context of communication reviews, designed to supplement the Cochrane Handbook [52] and related materials (e.g. Cochrane Training materials [73]) Developed in conjunction with editorial and statistical experts	Aim primarily to assist with understanding and applying specific review methods; they also intend, generally speaking, to prompt authors to improve reporting of the decisions made while performing the review Understanding, applying review methods, for example: • Cluster RCTs • Identifying comparisons • Heterogeneity and subgroup analysis • Meta-analysis • Narrative data synthesis and analysis
	Guides to support use of specific, complex methods	Guides to support implementation of specific methods, developed in response to MECIR [55] and in areas where major learning gaps or uncertainties were identified Developed in conjunction with editorial and methodological experts	To help with applying complex methods to reviews of complex interventions, and to improve reporting both of the methods used and decisions made; they also aim to support authors interpret their findings Applying methods, for example: • How to GRADE • Summary of findings • Describing results Reporting review methods, for example: • Describing methods and decisions adequately for replication • Acknowledging changes from protocol methods, including changes to selection criteria Interpreting findings, for example: • Considering certainty of evidence alongside findings • Considering size, precision and direction of effects • Describing results consistently
Resources with a conceptual focus	Information to support conceptual development of complex communication reviews	Tools to support conceptual framing of interventions and outcomes related to communication, and to prompt authors to consider theoretical models underpinning the review question Resources developed in conjunction with editorial and methodological experts Conceptual papers resulted from research projects	To help with applying complex methods to the development of reviews of complex interventions • Taxonomy of outcomes • Intervention taxonomies: o Consumers’ medicine use interventions o Childhood vaccination communication interventions • Examples of theoretical models

Editorially, we have also encountered a series of more specific recurrent problems in protocols and reviews. These tended to reflect difficulties in applying advice to complex communication reviews, such as ‘how to’ assess the suitability of analyses in cluster randomised trials, choose a meta-analysis model, select outcome(s) from many for meta-analysis and/or synthesis, organise the results according to the review’s objectives and major comparisons, explore heterogeneity, conduct narrative synthesis, or how to include a theoretical basis or model to explain the links between intervention and outcomes.

Such problems led us to develop a series of more specific resources (Table 1). These aim to assemble advice on a particular problem area within a single resource, and range from short supplemental guides for discrete methods, such as unit of analysis issues in cluster trials, guides for applying and reporting more complex methods such as GRADE in the context of complex interventions, and information to support conceptual development of protocols and reviews such as the outcomes and interventions taxonomies [59–61].

Many of these resources drew heavily on core methods from the Cochrane Handbook [52], but which authors had consistently shown difficulty implementing appropriately. In some cases, this may be because of the difficulties in applying technical information dispersed across sections of the Handbook, particularly where authors are not highly experienced systematic reviewers. In other cases, the problems we observed reflected unanticipated challenges with applying the Handbook and related methods guidance to complex interventions; for instance, the challenges associated with interpreting standardised mean differences, common in CCCG reviews, to make sense of the importance and size of effects.

While core methodological advice from the Cochrane Handbook and groups such as GRADE formed the basis for the CCCG resources, we needed to include additional information to help authors to interpret and apply the advice in the context of complex interventions, and to provide worked examples and practical instructions to help authors to deal with the complex decisions they routinely encountered. For other methods, such as undertaking narrative synthesis, which is common in CCCG reviews, advice has been missing or delayed in development, and so we needed to fill gaps to support authors to more readily complete reviews to a high standard.

In identifying problems, we found that these commonly fell into one or more of three broad stages of the review process, these being (1) understanding and applying systematic review methods, (2) reporting the review’s methods, and (3) interpreting the findings. Table 1 is annotated to show these stages with examples.

As well as the varying stages where problems become apparent, we recognise that different types of problems are often intertwined, with both practical issues of applying methods muddied by the conceptual problems associated with bringing order to a heterogeneous set of trials, comparisons, interventions and outcomes. Resources are developed with this awareness in mind as it is common that conceptual issues receive less attention in the development of systematic review methods. For a breakdown of the practical and conceptual problems encountered in the editorial process please see Table 2, with common challenges linked to examples of the resources developed to meet each. It is noteworthy that the problems listed here – both practical and conceptual – are typical of complex interventions.

**Table 2:** Common practical and conceptual challenges and examples of resources to address these

Type of challenge		Examples of resource^a^
Practical	Inconsistent language, no consensus on definitions, multidisciplinary	• Protocol template with guidance • Taxonomy of relevant outcomes • Describing results document • How to GRADE document
	Disparate data, often unsuitable for statistical pooling	• Meta-analysis document • Narrative data synthesis and analysis guide • Heterogeneity and subgroup analysis document
	Large size (many included studies)	• Data extraction template • Review template with guidance
	Many outcomes, measured in many different ways	• Taxonomy of relevant outcomes • Protocol template with guidance • Summary of findings document
Conceptual	Scope of review questions broad (lumped) or narrow (split)	• Protocol template with guidance
	Intervention purpose and delivery highly variable	• Protocol template with guidance • Data extraction template
	Comparisons complex and difficult to identify	• Identifying comparisons document
	Causal pathways or mechanisms socially complex or unknown	• Examples of theoretical models document • Taxonomy of relevant outcomes

^a^All resources are available at http://cccrg.cochrane.org/author-resources

Below we provide a short commentary on how the resources address key problem areas.

1. Understanding and applying systematic review methods

Key problems in applying systematic review methods include inconsistency in RoB ratings and ensuring authors use a transparent process for selecting outcomes for analyses, particularly when confronted by a plethora of incomparable outcomes at Review stage. The Protocol and Review templates have assisted in these areas because standardised text provides a practical framework for judging RoB domains transparently and consistently. Similarly, these templates provide a framework for outcome identification and selection to follow or adapt and this is linked with methodological guidance that addresses a range of common scenarios with examples of how each could be addressed.

Forming and operationalising comparisons is major problem area where generic advice has often been inadequate. In communication reviews, for example, intervention components may be difficult to disentangle from co-interventions, usual care may be highly variable, and isolating ‘active’ components to make reliable comparisons across studies can be challenging. What is planned at Protocol stage may be unworkable in the context of the trials included, particularly in large reviews. Specific guidance for identifying meaningful and justifiable comparisons is a resource which aims to focus authors on the questions(s) the review set out to address, what comparisons have been found versus what was sought, and how to group these for the purposes of analysis and synthesis.

We also found it necessary to develop detailed advice on ‘How to GRADE’ and ‘Preparing SoF tables’ because most author teams were unable to apply existing advice to communication reviews. Both of these documents aim to provide practical, stepwise guidance to help authors to develop SoF tables and apply GRADE ratings, with point-by-point prompts about the judgements needed at each stage and examples of how both the methods and outputs can be incorporated into reviews.

2. Reporting the review’s methods

Reporting is a key component of MECIR but challenging in the context of fluidity and the iterative nature of conducting a large and complex review. Additionally, many CCCG reviews do not contain data that is able to be meta-analysed and guidance on narrative synthesis methods and reporting has been slow to develop. We often found that there was little to no reporting of the rationale for grouping studies for comparison, analysis or data for synthesis. We addressed these issues firstly through the generic Protocol and Review templates and then through the Data extraction template, all of which encourage collection and explicit reporting of information underpinning the decisions made throughout the review’s conduct. Some of the specific resources developed, such as the narrative synthesis guidance, also prompt authors to more transparently report details associated with specific methods, or the Taxonomy of outcomes which can be used to improve consistency of language in conceptually complex areas, although improving reporting is not the primary aim of these resources.

3. Interpreting the findings

Key problem areas are interpreting the findings in conjunction with applying GRADE, preparing SoF tables and describing results within reviews. All led to resources which extended the advice available to focus on the practical application of the methods and the decisions required by authors. As a collection, these resources largely focus on supporting authors to consider the certainty of evidence alongside the findings, and to explicitly prompt authors to consider the size, precision and direction of effects – considerations which we had found were commonly missing in CCCG reviews. This is with the overarching aim of being able to accurately and consistently describe, and to attach meaning, to the review’s findings. For more complex problems such as common or core outcomes in the communication research literature, we have involved staff in empirical research to develop taxonomies of interventions and outcomes which may provide a consistent benchmark for authors undertaking reviews in these areas [59–61].

It should be noted that our editorial assessment of common issues in protocols and reviews was not formalised, and so we did not count the frequency of each type of problem encountered other than in editorial discussions, where we noted the issues we were seeing frequently. This was due to the evolving nature of our editorial processes, which naturally became more directed towards the identification of major issues over time. It was also due to the multiplicity of problems we often identified within each protocol or review. For example, the problem of how to deal with disparate data not suited to statistical pooling was often compounded by inclusion of highly varied interventions (and comparator groups), assessed with multiple incongruent outcome measures. Such problems are not independent of one another and our focus was therefore on the interrelationships between these problems rather than the frequency with which they occurred.

We did not formally evaluate the resources, principally because it was expected that the updated Cochrane Handbook would address many of the issues that had been recognised for several years [10, 11]. However, the production of the Handbook took longer than anticipated and so the opportunity for evaluation was missed. We have, however, recently started collecting download figures and these indicate consistent use of the resources over the period since July 2018 (Table 3). Numbers of downloads indicate that general resources, such as the templates, and more specific guidance, like the ‘How to GRADE’ guide and those on narrative synthesis, are all well accessed.

**Table 3:** Number of downloads for Cochrane Consumers and Communication Group (CCCG) author resources (July 2018 to 4 March 2019)

Resource	Times downloaded
Protocol template with guidance	156
Review template with guidance	127
Data extraction template	204
Cluster randomised controlled trials	75
Equity	44
Examples of narrative synthesis	152
Examples of theoretical models	63
Identifying comparisons	61
Heterogeneity and subgroup analysis	102
Meta-analysis	95
Narrative data synthesis and analysis	131
Summary of findings	135
How to GRADE	260
Describing results	153
Study design guide	84
Study quality guide	125
Taxonomy of relevant outcomes	67

## Discussion

The CCCG resources are designed to be used in tandem with the Cochrane Handbook and other existing bodies of methodological advice, and aim to address recurrent problems that our authors faced when applying this more standardised advice to reviews on complex communication interventions. The range of methodological problems identified has been considerable. Whilst the standardised Cochrane advice is wide-ranging in the examples used, it has required considerable tailoring to provide enough detail to assist authors to interpret and apply the advice in the context of the complex communication research literature. Often, this meant including case examples to help authors work through the complexities and implications of decisions made in their reviews. In practice, this means that the role of the CCCG resources, aside from filling gaps in guidance where these are apparent, is to serve as a bridge between the standardised theoretical advice available and its implementation in the conduct of reviews (what authors do), and as such the aim is to provide practical, workable advice drawing from others’ extensive methodological expertise.

Over time, we have found improvements in several areas that were previously highly problematic. For instance, use of the Protocol template has vastly improved the reporting of decisions related to setting up selection criteria, such as which comparisons the review will assess, and processes for selecting outcomes and outcome measures for analysis. Since the template contains standardised wording for authors to adapt, it has also led to major improvements in the coherence and comprehensiveness of the description of methods as a whole, which were previously highly problematic. These improvements in methods carry forward to the review stage and have enabled our editorial focus to shift from major recurrent corrections of the description of methods to one that concentrates on conceptual coherence and clarity. The Review template too has greatly improved the transparency of reporting in our reviews. Prompts to authors and examples provided in the template have helped to promote clear reporting of decisions about the approaches to synthesis, groupings of studies, and whether or not to statistically pool data. Providing examples of common problems and issues to consider in relation to the data itself (numerical and descriptive data, as well as summary statistics) have also improved the organisation and reporting of findings within reviews. Such improvements, amongst others, have helped to shift the editorial focus from accurately describing results to supporting authors in interpreting the meaning of findings and in developing consistent messages about these.

### Lessons for editorial practice

Viewing the last several years, the development and updating of general and targeted methodological resources has highlighted some key lessons for us as an editorial team. These include:

- Disaggregating complex methods into step-by-step processes, with prompts to assist authors to work through decisions at each step, appears useful and is reflected in improved application and reporting of methods in CCCG reviews over time.
- Providing examples of the application of methods and good practice can help authors to apply complex information in the context of their own work and may be amongst the most valuable information for those immersed in conducting reviews. This is particularly important when the core methods focus on clinical scenarios or data, as much of the research on complex communication interventions does not conform to the standard clinical model; these challenges are well-recognised for complex interventions [3, 4].

The resources needed to develop these additional supports for authors, and to keep them up-to-date as methodological advice or standards change, are also considerable. We stay abreast of methodological developments within and outside Cochrane, and continuously check the acceptability and usability of the resource collection as protocols and reviews are submitted to the editorial base. When sufficient new methods become available, or we notice consistent difficulties with use of a particular resource, we identify whether, and how, the resource might be changed and updated. The updating of the resources therefore happens on an as-needed basis.

The CCCG actively promotes the resources within Cochrane networks, with the aim of reducing duplication of efforts to support authors where resources (or adapted versions) may be useful to others. It is also hoped that the updated edition of the Cochrane Handbook, which will soon be fully available, will reduce the need for such additional guidance. For instance, new chapters, such as those on synthesising findings using methods other than meta-analysis, fill a long-standing gap in available advice for authors, and such new methodological advice is likely to supersede and replace some CCCG resources. However, our experience at the ‘how to implement methods’ end of the spectrum suggests that challenges will remain, and that there will likely be continued need for practical advice and worked examples from which authors can work to find solutions to the challenges raised by complex interventions. The implied broad-ranging educational purpose of the Cochrane Handbook model may also need further consideration in the context of continually evolving methods and their implementation.

The demands of meeting methodological standards for systematic reviews, and the inevitable variation in implementation of methods [62], is not described only for Cochrane [55–57]; even in large, well-established programmes such as the Agency for Healthcare Research and Quality Evidence-based Practice Center difficulties meeting required standards have been described, and the need for tailored advice to help people to implement the overarching methodological principles has been highlighted [63]. This may have implications for how new review methods are implemented by organisations in the future.

### Lessons for editorial policy and research

Health communication is a rapidly growing research area, at least in part because of demand for information on evidence-based strategies from policy-makers, safety agencies and health services. Rapid growth presents an array of significant ongoing challenges. For example, the rapidly evolving terminology and proliferation of different communication interventions not only present authors and editors with difficulties, but it will make the task of commissioning or prioritising new research similarly complex. Health services may be under pressure to implement new modalities but it is not always clear how they differ from older forms.

Our editorial response, along with many Cochrane Groups, has been to revise our updating policy [64, 65] and develop new social research methods for clearly defining and prioritising review questions with input from a range of stakeholders [66]. Others have highlighted the need for research to understand systematic reviews of complex interventions more broadly, for instance, to extend methods and review questions to more meaningfully account for complexity in the context of implementation within complex contexts and systems [67]. It is also possible that closer integration of policy-makers and users of research into research generation and conduct will lead to further demands on systematic reviewers. These could include demands for different questions, different methods [68] or a move towards considering the implementation context for complex intervention reviews more explicitly.

## Conclusions

Policy-makers and other decision-makers increasingly want and rely on information about evidence-based health communication strategies to inform decisions about safe, high-quality healthcare. Systematic reviews of the effects of communication strategies provide a key component of the evidence base for such decisions, but practical and conceptual challenges contribute to problems in reviews of complex communication interventions. Identifying common problems and developing resources to fill gaps or to serve as a bridge between the theory and practice of reviews in complex areas can help to support the implementation of Cochrane review methods.

## Abbreviations

CCCG: Cochrane Consumers and Communication Group
EPOC: Cochrane Effective Practice and Organisation of Care Group
GRADE: Grading of Recommendations Assessment, Development and Evaluation system
MECIR: Methodological Expectations for Cochrane Intervention Reviews
RoB: risk of bias
SoF: summary of findings
TIDieR: Template for intervention description and replication
